# Supplementary material for: Modulation of proteoglycan receptor PTPσ enhances MMP-2 activity to promote recovery from multiple sclerosis
Source: Nat Commun. 2018 Oct 8;9:4126. doi: 10.1038/s41467-018-06505-6 (PMC6175851; doi:10.1038/s41467-018-06505-6)
Supplement: Supplementary file 1 — Supplementary Information [file 41467_2018_6505_MOESM1_ESM.pdf]

Modulation of the proteoglycan receptor PTPσ enhances MMP-2 activity to promote recovery from multiple sclerosis

Luo et al.

Supplementary Figure 1

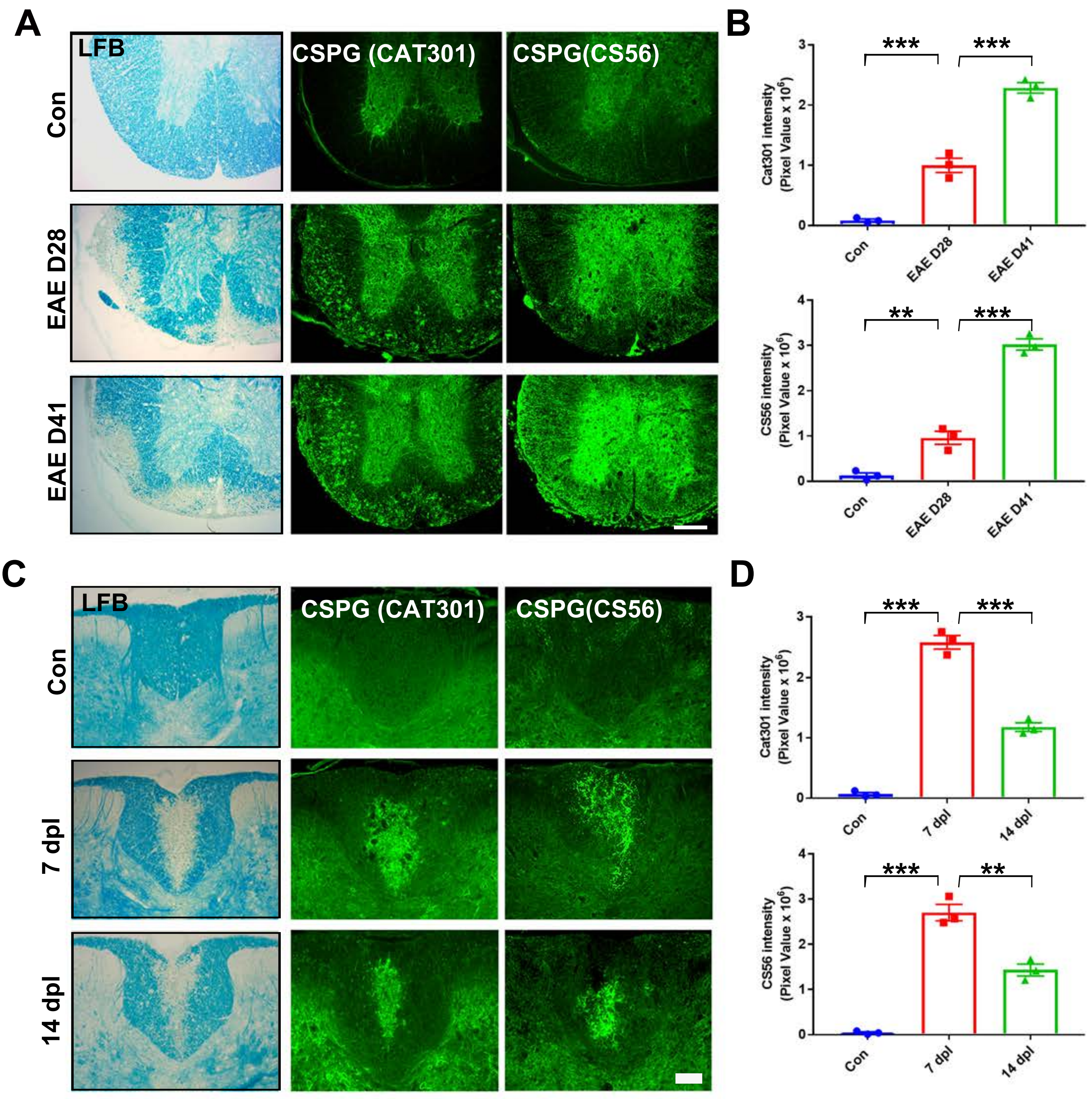

Supplementary Figure 1: increased CSPG load in mouse models of MS.

A-B. Representative LFB-stained sections and immunohistochemistry images of Cat301 and CS56 show CSPG accumulation in the thoracic spinal cord of EAE mice at 28 and 48 days post-induction. Scale bar=100 µm. Quantification of pixel intensities of Cat301 (aggrecan CSPG) and CS56 (glycosaminoglycan moieties of CSPGs) depicted (Cat301: n=3 mice/group, ANOVA $F(2,6)=163.9$, Tukey's multiple comparison test, $P_{\text{con versus EAE D28}} = 0.0007$, $P_{\text{EAE D28 versus EAE D41}} = 0.0001$; CS56: n=3 mice/group, ANOVA $F(2,6)=168.7$, Tukey's multiple comparison test, $P_{\text{con versus EAE D28}} = 0.0052$, $P_{\text{EAE D28 versus EAE D41}} < 0.0001$). **C-D.** Representative LFB-stained sections and immunohistochemistry images of Cat301 and CS56 show CSPG accumulation in the lesion site after LPC demyelination at 7dpl and 14dpl. Scale bar=100 µm. Quantification of pixel intensities of Cat301 and CS56 depicted (Cat301: n=3 mice/group, ANOVA $F(2,6)=269.7$, Tukey's multiple comparison test, $P_{\text{con versus 7 dpl}} < 0.0001$, $P_{\text{7dpl versus 14 dpl}} < 0.0001$; CS56: n=3 mice/group, ANOVA $F(2,6)=105$, Tukey's multiple comparison test, $P_{\text{con versus 7 dpl}} < 0.0001$, $P_{\text{7dpl versus 14 dpl}} = 0.0011$). The data are presented as mean±s.e.m. $*P<0.05$, $***\ p < 0.001$.

Supplementary Figure 2

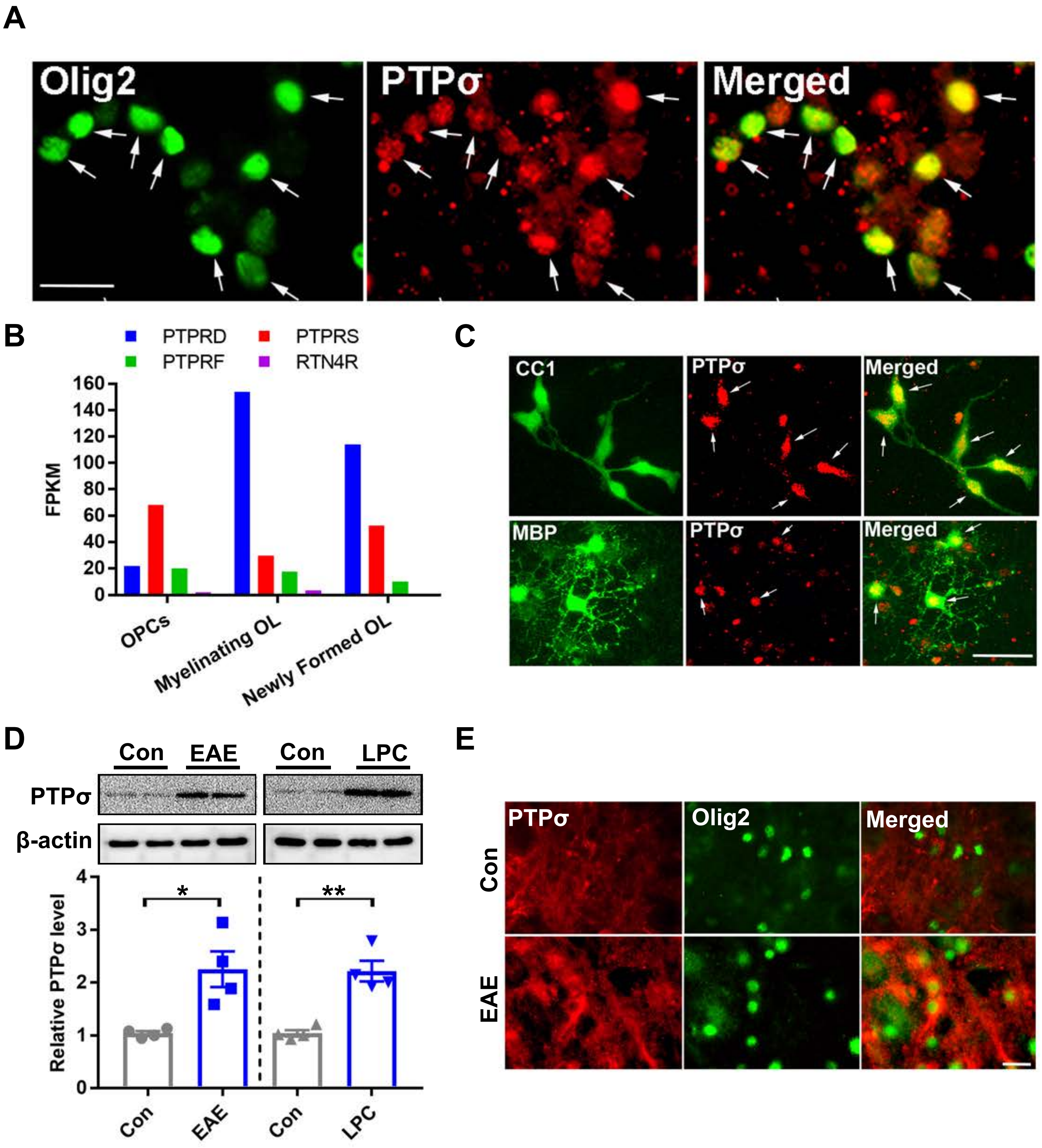

Supplementary Figure 2: PTPσ expression is enhanced following EAE and LPC.

A. Representative immunocytochemistry images of PTPσ and Olig2 show PTPσ expression on oligodendrocyte progenitor cells grown *in vitro.* Scale bar=50 μm. **B.** Graphical representation of cell-specific PTPRD (PTPδ), PTPRS (PTPσ), PTPRF (LAR) and RTN4R (Nogo) mRNA levels obtained from a publicly available RNA-sequencing transcriptome database [https://web.stanford.edu/group/barres_lab/]. FPKM represents fragments per kilobase of transcript sequence per million mapped fragments. **C.** Representative immunohistochemistry images of PTPσ, CC1, and MBP show PTPσ expression in $CC1^+$ or MBP^+ cells in OPCs culture in vitro. Scale bar=50 μm. **D.** Western blot analysis of PTPσ expression in the thoracic spinal cord of EAE or LPC-induced demyelination mice. Data are normalized to β-actin protein expression (n=4 mice per group. $P_{\text{con versus EAE}}$ =0.012, t=3.558, df=6; $P_{\text{con versus LPC}}$=0.0012, t=5.775, df=6; two-tailed unpaired Student's t test). **E**. Representative immunohistochemistry images of PTPσ and Olig2 show increased expression of PTPσ in $Olig2^+$ cells in the spinal cord of EAE mice at 28 days post-induction. Scale bar=50 μm. The data are presented as mean±s.e.m. *P<0.05, **P<0.01.

Supplementary Figure 3

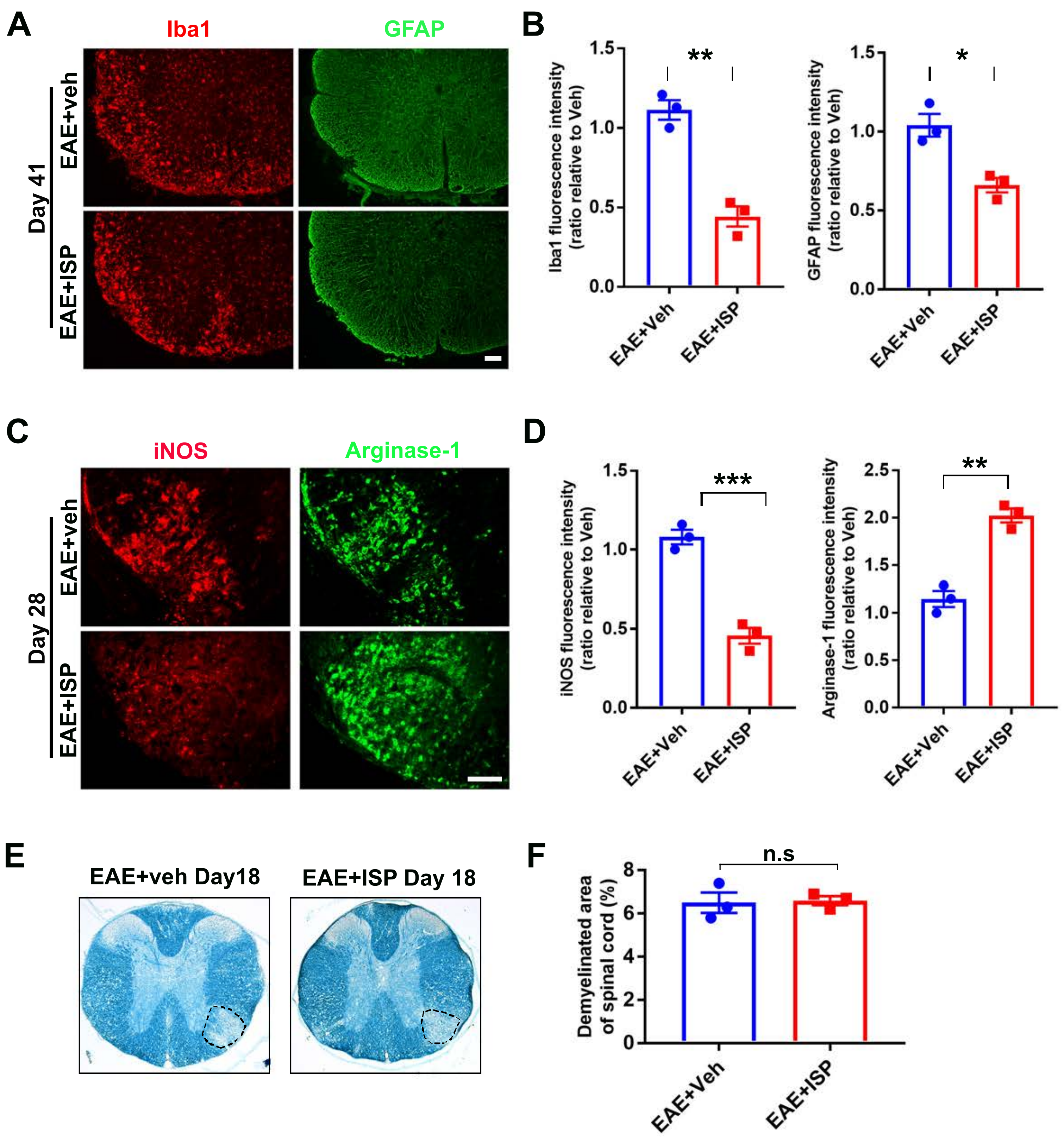

Supplementary Figure 3: ISP modulates inflammation in EAE models in mice.

A. Representative immunohistochemistry images of Iba1 and GFAP show decreased activation of microglia and astrocytes respectively in the spinal cord of ISP-treated mice at day 41 following EAE induction. Scale bar= 100 μm. **B.** Relative quantification of immunofluorescence intensity of Iba1 and GFAP in the spinal cord of ISP-treated mice at day 41 (n= 3 mice/group， Iba1: P=0.0016, t=7.608, df=4; GFAP: P=0.0113, t=4.448, df=4. two-tailed unpaired Student's t test). **C.** Representative immunohistochemistry images of iNOS (M1 microglia marker) and Arginase-1 (M2 microglia marker) show increased M2 microglia and decreased M1 microglia in the spinal cord of ISP-treated mice at day 28. Scale bar= 100 μm. **D.** Quantification of relative immunofluorescence of iNOS and Arginase-1 in the spinal cord of ISP-treated mice at day 41 (n= 3 mice/group， iNOS: P=0.0008, t=9.114, df=4; Arginase-1: P=0.0014, t=7.824, df=4. two-tailed unpaired Student's t test). **E.** Representative Luxol fast blue staining images show no differences in demyelination area of Vehicle or ISP-treated mice at day 18. **F.** Quantification of demyelination area in the spinal cord of Vehicle or ISP-treated mice at day 18 indicates similar levels of demyelination in EAE model (n= 3 mice/group， P=0.8559, t=0.1936, df=4, two-tailed unpaired Student's t test). The data are presented as mean±s.e.m. *P<0.05, *** p < 0.001, n.s: no significance.

Supplementary Figure 4

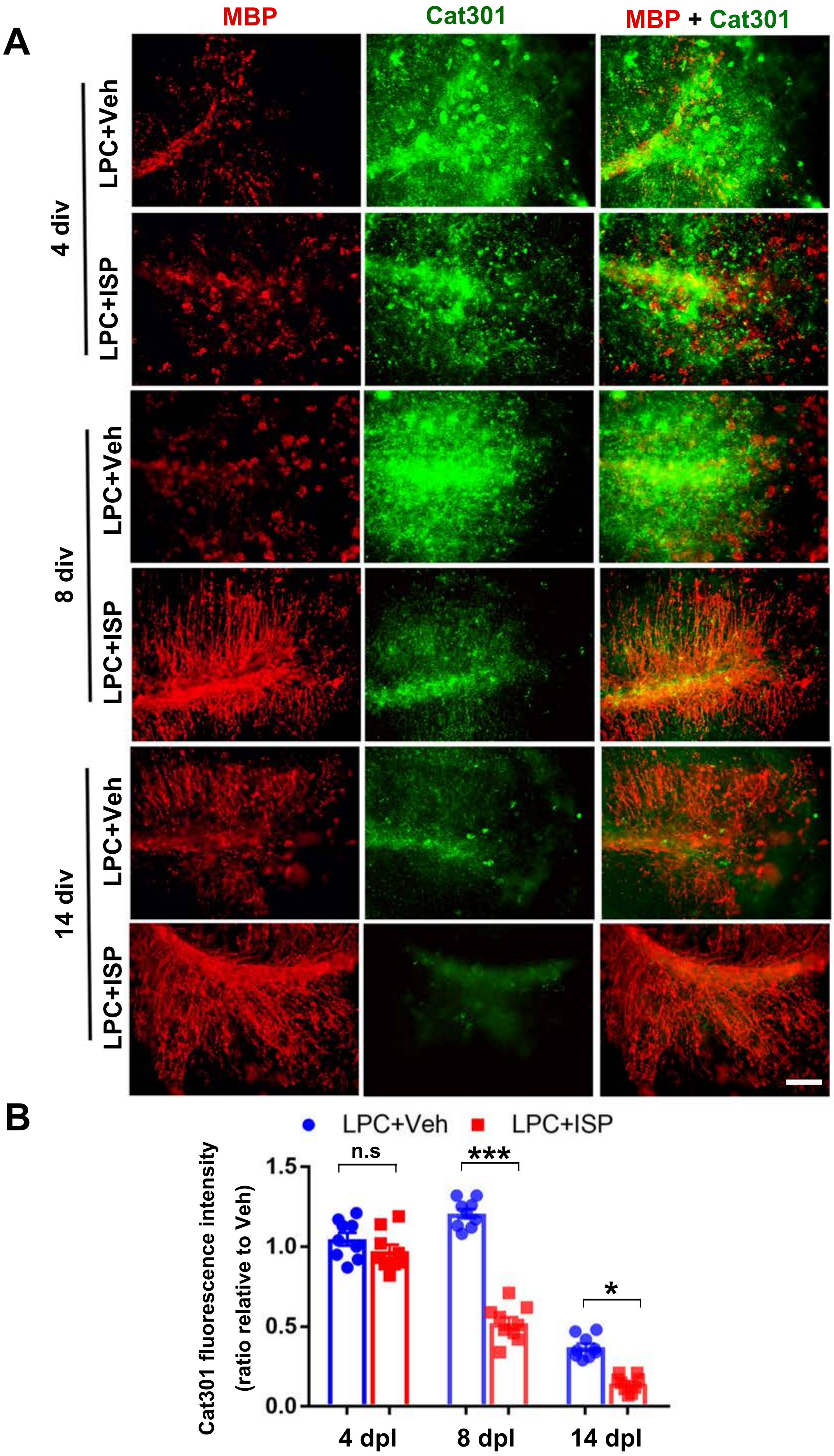

Supplementary Figure 4: ISP increases CSPG clearing as remyelination occurs.

A. Representative immunohistochemistry images of MBP and Cat301 show reduced abundance of CSPG after ISP treatment in LPC-demyelinated cerebellar slices at 8dpl and 14dpl. Scale bar=100 μm. **B.** Relative quantification of immunofluorescence intensity of Cat301 after Vehicle or ISP treatment in LPC-demyelinated cerebellar slices at 4dpl, 8dpl and 14dpl (n=9 slices from 3 independent replicates per group, two-way ANOVA $F(2,6)=30.78$, Sidak's multiple comparison test, 8dpl: $P_{\text{LPC+Veh versus LPC+ISP}} < 0.0001$, 14dpl: $P_{\text{LPC+Veh versus LPC+ISP}} = 0.0325$). The data are presented as mean±s.e.m. *$P<0.05$, *** $p < 0.001$, n.s: no significance.

Supplementary Figure 5

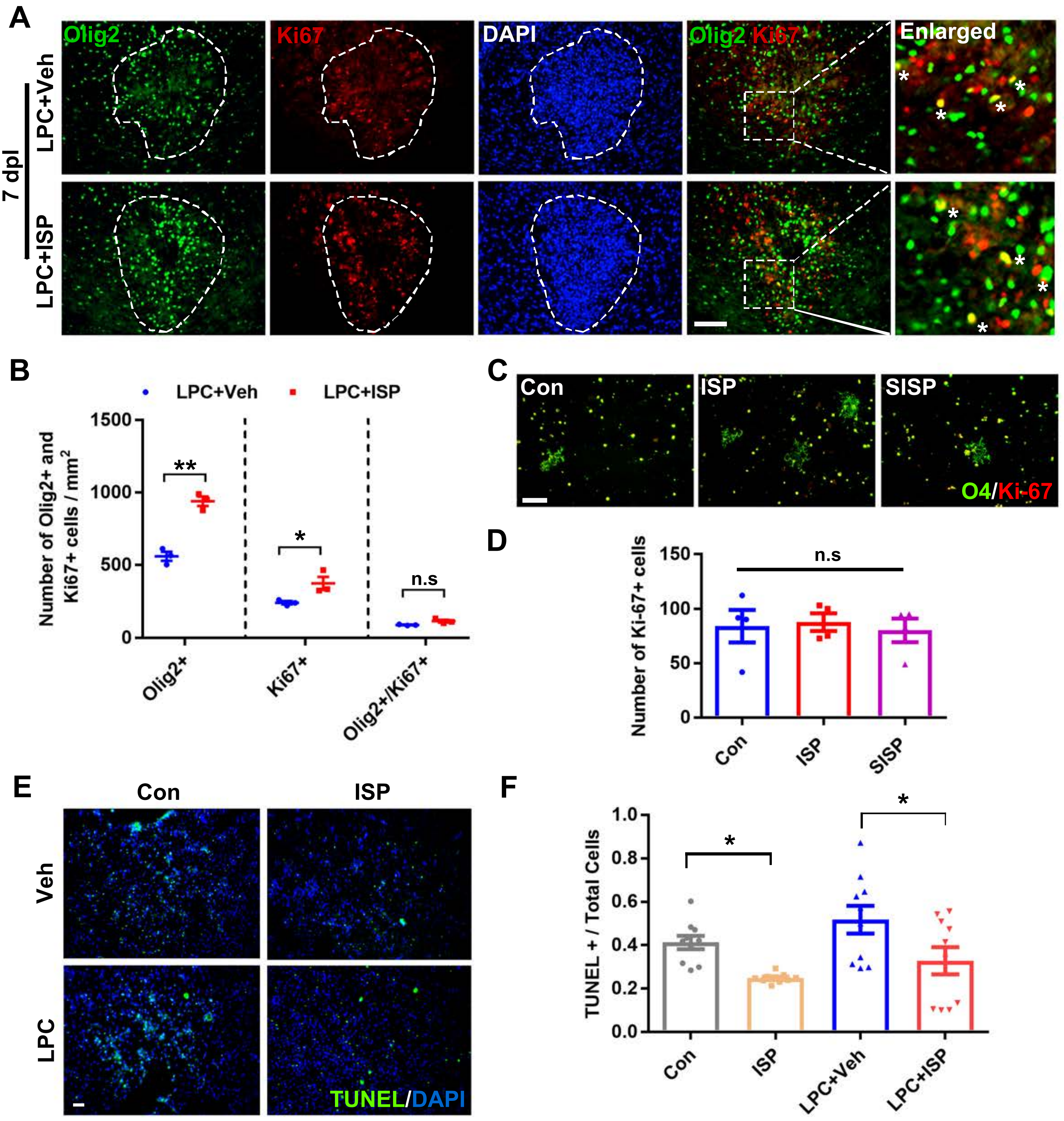

Supplementary Figure 5: ISP promotes OPC recruitment and survival on CSPGs.

A. Representative images of Ki67 immunostaining showing proliferating OPCs ($Olig2^+$) in the spinal cord of vehicle- and ISP-treated mice at 7dpl. White arrows show $Olig2^+/Ki67^+$ cells. Scale bar=100 μm. **B.** Quantification of immunostaining showing $Olig2^+$ cells/mm^2, $Ki67^+$ cells/mm^2 and $Olig2^+/Ki67^+$ cells/mm^2 in the spinal cord of vehicle- and ISP-treated mice at 7dpl. (n=3 mice/group, Olig2: *P*=0.0012, t=1.6, df=4; Ki67: *P*=0.0449, t=2.883, df=4; $Olig2^+/Ki67^+$: *P*=0.056, t=2.666, df=4. two-tailed unpaired Student's t test). The data are presented as mean±s.e.m. **C.** OPCs were cultured on aggrecan/laminin-precoated coverslips, treated with vehicle control, 2.5uM ISP or SISP, and stained with O4 and Ki67. Scale bar=100 μm. **D.** Ki67 was not significantly changed among the groups (One-Way ANOVA, Tukey's posthoc test, P=0.8998, F(2,9)=0.1068, N=12 images each with 4 replicates and 3 repeats each). **E.** OPCs were cultured on aggrecan/laminin and treated with control or 2.5uMISP for 2div before incubation with vehicle or LPC (1ug/mL) for 2 hours before DAPI and TUNEL staining. Scale bar=100 μm. **F.** Quantification of $TUNEL^+$ over $DAPI^+$ cells reveal significant survival of ISP-treated (N=40 images, 4 replicates) OPCs over control (One-Way ANOVA, Tukey's posthoc test, P=0.0021, F(3,36)=5.96). ISP-treatment enhanced survival of LPC-treated cells (N(images)=36 control, 28 each LPC-treated group). The data are presented as mean±s.e.m. *P<0.05, **P<0.01, *** p < 0.001.

Supplementary Figure 6

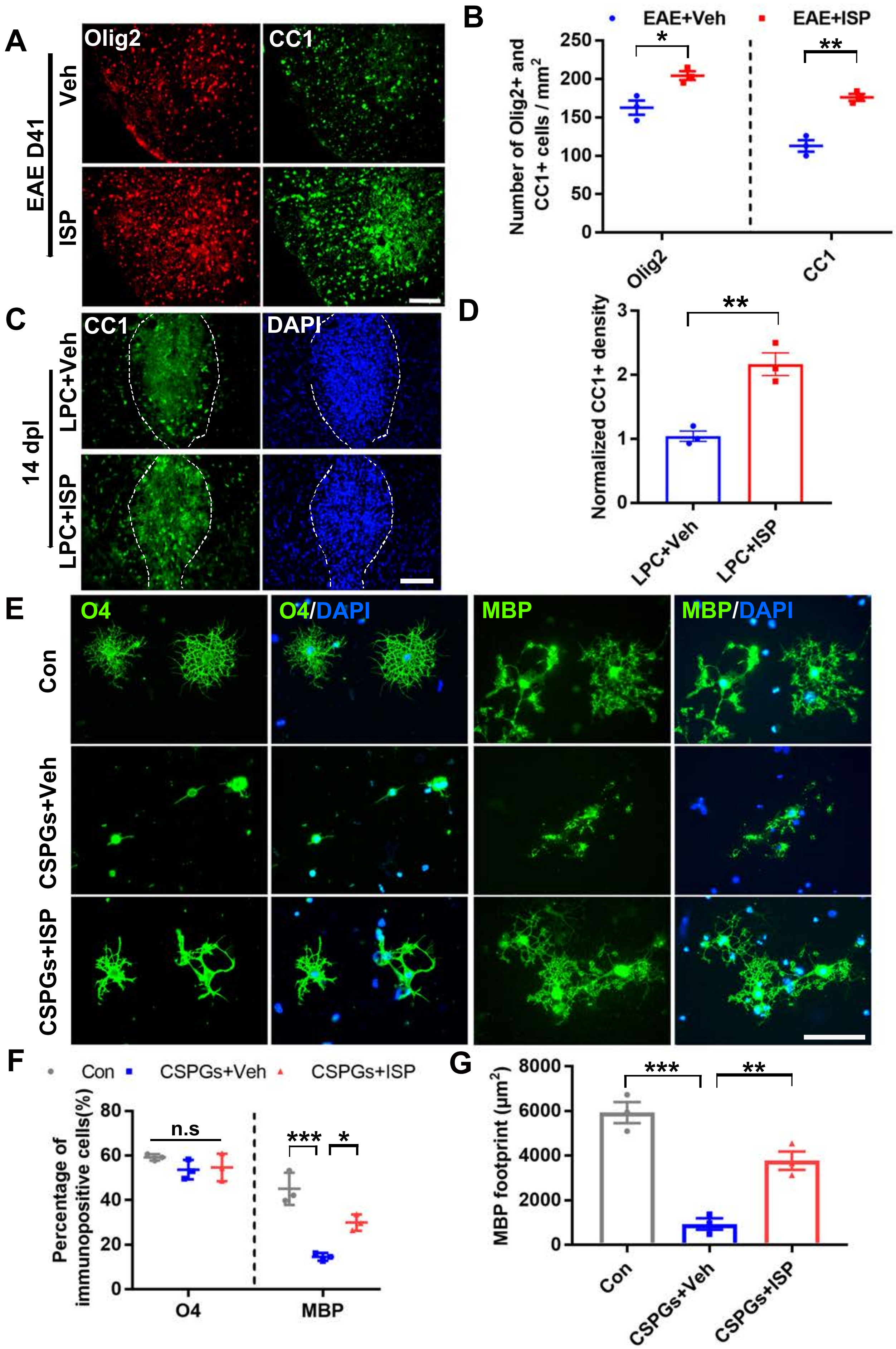

Supplementary Figure 6: ISP enhances OPC process outgrowth and maturation.

A. Representative immunohistochemistry images of Olig2 and CC1 in the thoracic spinal cord of vehicle or ISP-treated EAE mice at 41 days post-induction. Scale bar=50 μm. **B.** Quantification of immunostaining for Olig2^{+} cells/mm^{2} and CC1^{+} cells/mm^{2} in the thoracic spinal cord of vehicle or ISP-treated EAE mice at 41 days post-induction (n=3 mice/group, Olig2: *P*=0.0189, t=3.811, df=4; CC1: *P*=0.0019, t=7.259, df=4. two-tailed unpaired Student's t test). **C.** Representative immunohistochemical images of DAPI and CC1 in the spinal cord of vehicle or ISP-treated LPC mice at 14dpl. Dashed lines demarcate lesion areas. Scale bar=100 μm. **D.** Quantification of immunostaining for normalized CC1^{+} oligodendrocytes density at 14dpl (n=3 mice/group, *P*=0.0044, t=5.789, df=4. two-tailed unpaired Student's t test). **E.** Representative immunohistochemical images of the maturation of OPCs after plating onto poly-l-lysine or CSPGs in the presence of ISP or vehicle. Scale bar=100 μm. **F.** Quantification of the relative proportion of maturing OLs after OPCs plating onto poly-l-lysine (control) or CSPGs in the presence of ISP or vehicle (n= 3 independent replicates, O4: ANOVA F(2,6)=1.321, P=0.3347; MBP: ANOVA F(2,6)=30.99, Tukey's multiple comparison test, $P_{\text{Con versus CSPGs+Veh}}$ = 0.0005, $P_{\text{CSPGs+Veh versus CSPG+ISP}}$ =0.0175). **G.** Comparison of the size of MBP^{+} footprints after OPCs plating onto poly-l-lysine (control) or CSPGs in the presence of ISP or vehicle at 6 days (n= 3 independent replicates, ANOVA F(2,6)=40.96, Tukey's multiple comparison test, $P_{\text{Con versus CSPGs+Veh}}$ = 0.0003, $P_{\text{CSPGs+Veh versus CSPGs+ISP}}$ =0.0052). The data are presented as mean±s.e.m. *P<0.05, **P<0.01, *** p < 0.001. n.s., not significant.

Supplementary Figure 7

A

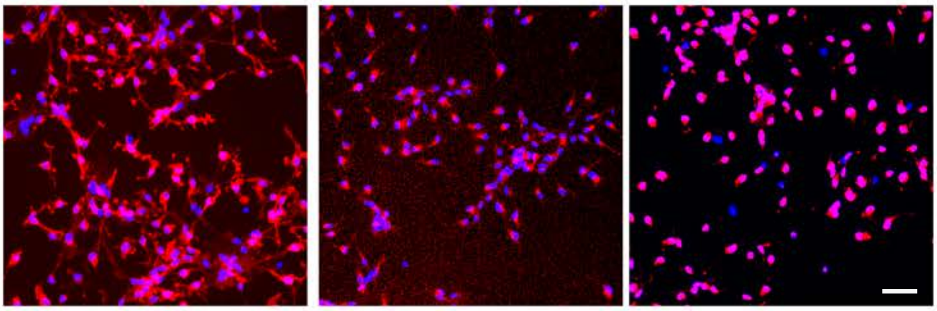

NG2+ cells: 96.2%

PDGFRα+ cells: 95.8%

Olig2+ cells: 95.2%

B

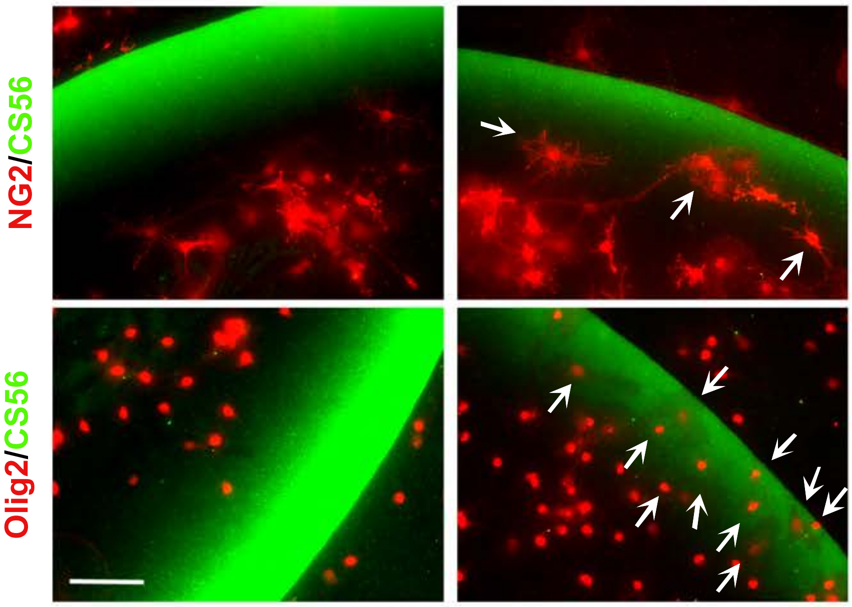

C

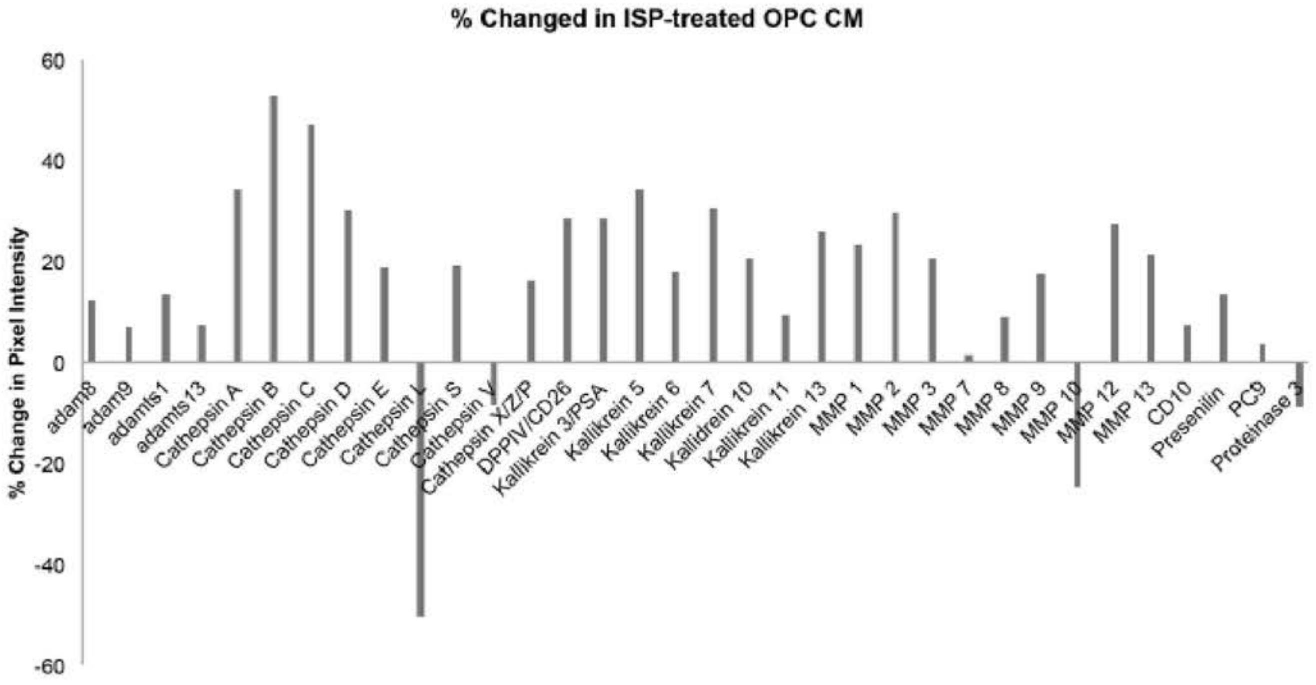

Supplementary Figure 7: OPCs purity, migration and Protease Array.

A. Representative images of immunostaining of NG2, PDGFRα and Olig2 in purified OPCs cultures indicating the OPCs purity is >95%. Scale bar=100 μm. **B.** Representative images of immunostained NG2+, and Olig2+ OPCs (red) on CSPG (CS56, green) barrier depicting CSPG degradation after ISP treatment as OPCs cross the barrier (white arrows). Scale bar=50 μm. **C.** To begin screening which proteases may be upregulated by ISP treatment, cultured OPCs were treated with vehicle control or 2.5uM ISP for 4 days in vitro. Conditioned media was then incubated with qualitative protease array (R & D Systems) and developed. % Change in pixel intensities of ISP-treated vs. control OPC CM was then calculated.

Supplementary Figure 8

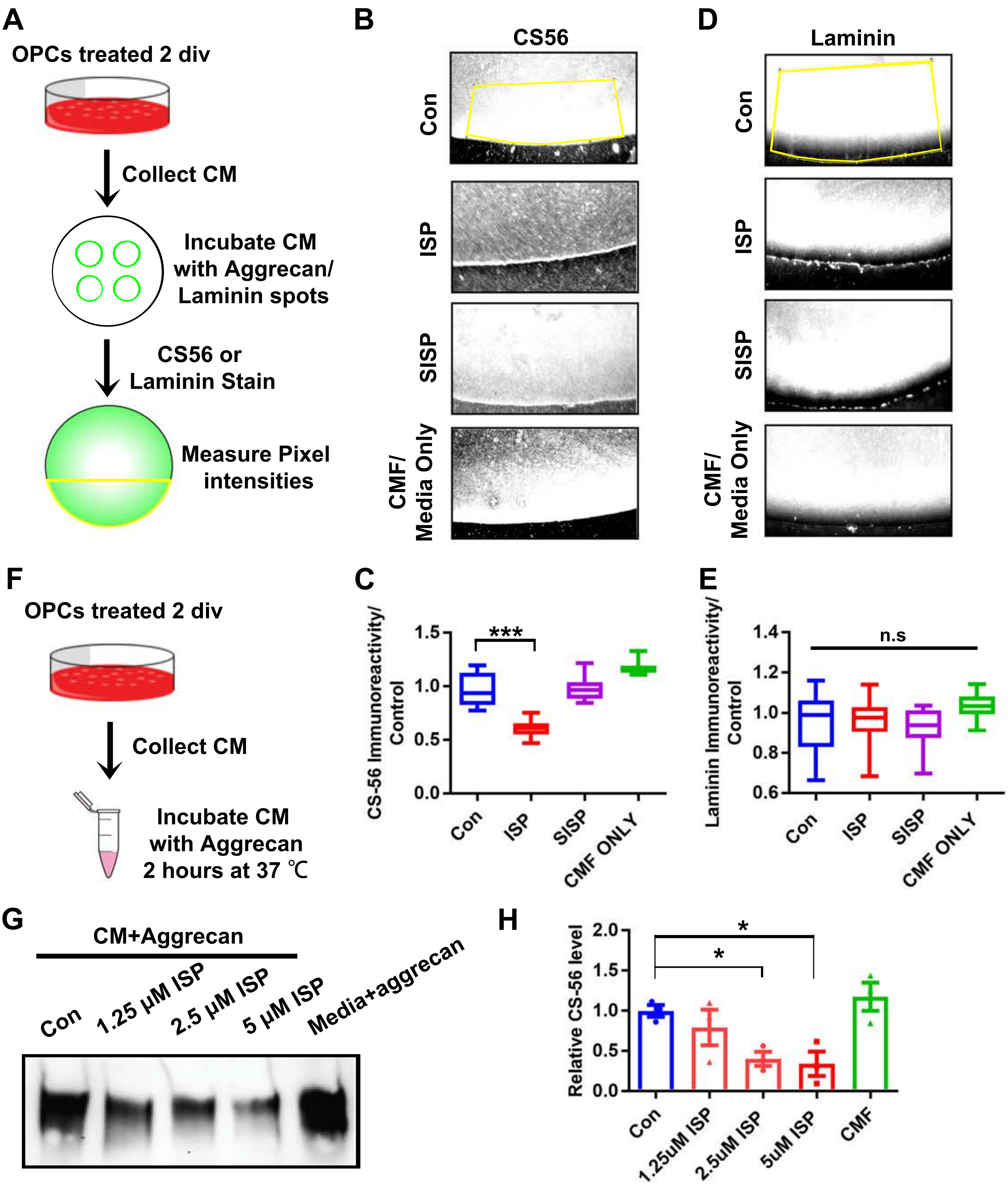

Supplementary Figure 8: ISP enhances CS56 degradation in a dose-dependent manner.

A. Diagrams illustrate the experimental design (Created by F.L. and A.P.T.). OPCs treated with vehicle control, 2.5uM ISP or SISP was incubated for 2 days in vitro before CM was collected and incubated with aggrecan/laminin spots. An additional subset of spots was incubated for the same duration with media only. Spots were immunostained with CS56 or laminin and the pixel intensities of the spot rim were recorded. Representative images of CS56-stained spots with yellow region of interest indicating measured portion of the spot. **B.** Representative images of CS56-stained spots showed that ISP-treated OPC CM is capable of degrading CS56 spots. **C.** Quantification of CS56-immunostained spot indicates ISP-treated OPC CM significantly degrades CSPGs over control (One-Way ANOVA, Dunnett's posthoc test, P=0.0001, F(5,72)=45.19). N(images, 5 replicates)=69 Control, 104 ISP, 95 ISP, 31 Media only). **D.** Representative images of laminin-stained spots. **E.** Quantification of laminin-stained spots indicate no significant changes between groups (One-Way ANOVA, Tukey's posthoc test, P=0.0818, F(3,85)=2.312), N(images, 5 replicates)=133 Control, 134 ISP, 114 SISP, 34 Media Only). **F.** Diagrams illustrate the experimental design for confirming CS56 degradation. OPCs were treated with varying doses of ISP or vehicle control and incubated with a fixed concentration of aggrecan (20ug/mL) for 2 hours before western blot analysis. **G.** Western blot analysis of CS56 and subsequent. **H.** Quantification of CS56 band indicate significant ISP-treated CS56 degradation over control at 2.5 and 5uM doses (One-Way ANOVA, Tukey's posthoc test, P=0.0049, F(5,12)=6.112, N=3 western blots). The data are presented as mean±s.e.m. *P<0.05, *** p < 0.001. n.s., not significant.

Supplementary Figure 9

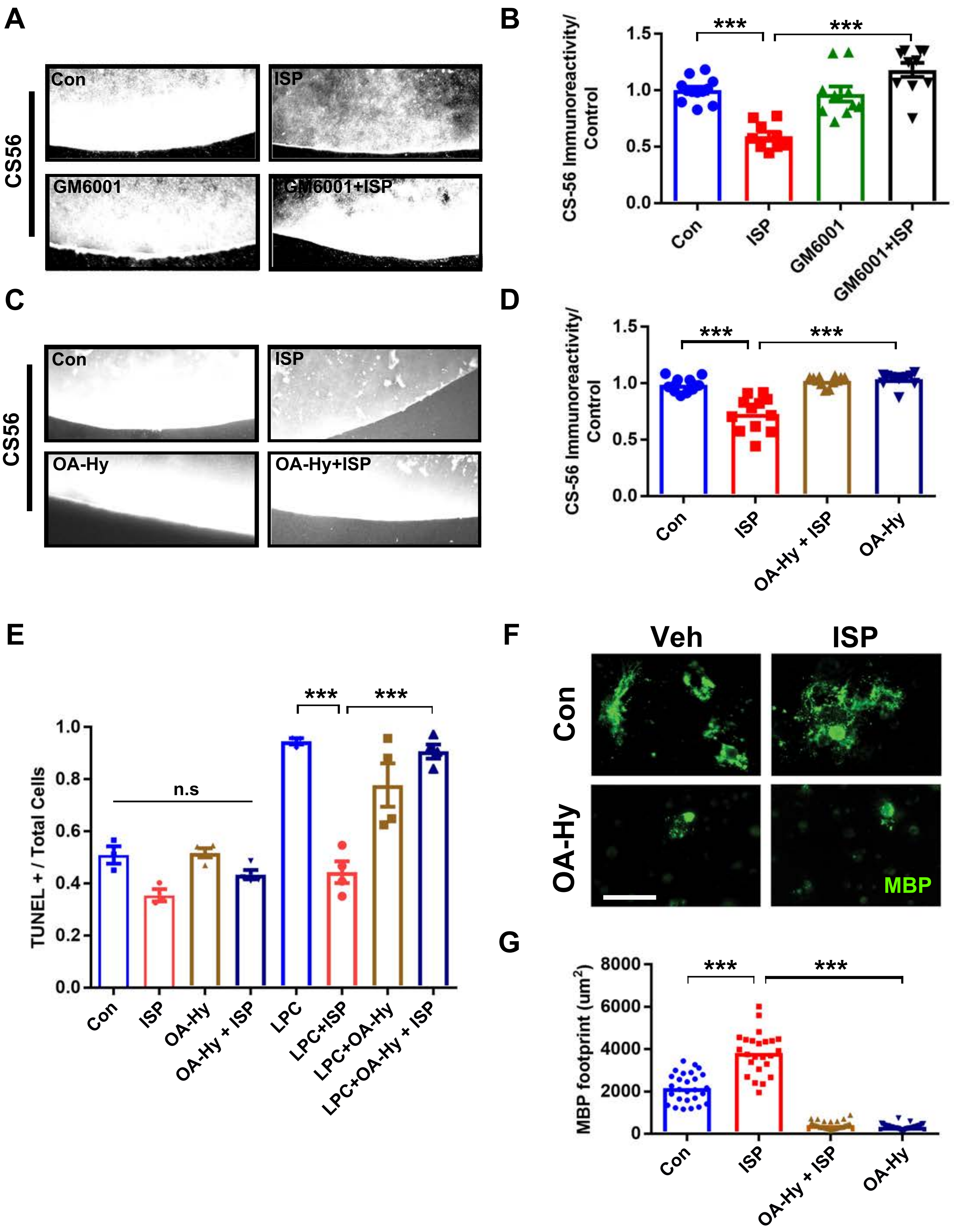

Supplementary Figure 9: Protease inhibitors attenuate ISP-induced CSPG degradation and subsequent CSPG-OPC disinhibition.

A-B. The functional effects of protease inhibitors on OPCs, CS56-immunostained spots were incubated with vehicle control, 2.5uM ISP, or 25uM GM6001 +/- ISP treated OPC CM. Quantified CS56 immunoreactivity reveals significant ISP-induced CS56 degradation over control and GM6001+ISP (One-Way ANOVA, Tukey's posthoc test, $P=0.0001$, $F (3, 37) = 21.43$), N(images, 3 replicates)=24 Control, 17 ISP, 19 GM6001, 20 GM6001+ISP). **C-D.** Aggrecan spots treated with vehicle control, 2.5uM ISP, or 100nM MMP-2 inhibitor (OA-Hy) +/- ISP revealed ISP-induced degradation over vehicle control and MMP-2 inhibitor (OA-Hy) + ISP (One-Way ANOVA, Tukey's posthoc test, $P=0.001$, $F (3, 44)=31.50$). N=24 images each). **E.** To assess whether the MMP-2 inhibitor affected apoptosis, OPCs cultured on aggrecan/laminin precoated coverslips were treated with ISP, 100nM MMP-2 inhibitor (OA-Hy) +/- ISP for 2 days in vitro. A subset of treated OPCs was additionally challenged with LPC (1ug/mL) incubation for 2 hours before TUNEL/DAPI staining. MMP-2 inhibitor (OA-Hy) did not significantly increase $TUNEL^+$ cells/total cells. However, MMP-2 inhibitor (OA-Hy) even with ISP treatment increased apoptosis following LPC treatment over LPC-ISP treatment (One-Way ANOVA, Tukey's posthoc test, $P=0.0001$, $F(7,21)=29.66$), N=2 replicates, 2 wells each). **F.** 100nM MMP-2 inhibitor (OA-Hy) treatment of OPCs cultured on aggrecan/laminin additionally decreased MBP+ footprints significantly over ISP treatment alone (One-Way ANOVA, $P=0.0001$, $F(3,112)=228.3$), N(cells, 5 replicates total)=128 Control, 126 ISP, 191 OA-Hy, 222 OA-Hy+ISP). Scale bar=100 µm. The data are presented as mean±s.e.m. $**P<0.01$, $*** p < 0.001$. n.s., not significant.

Supplementary Figure 10

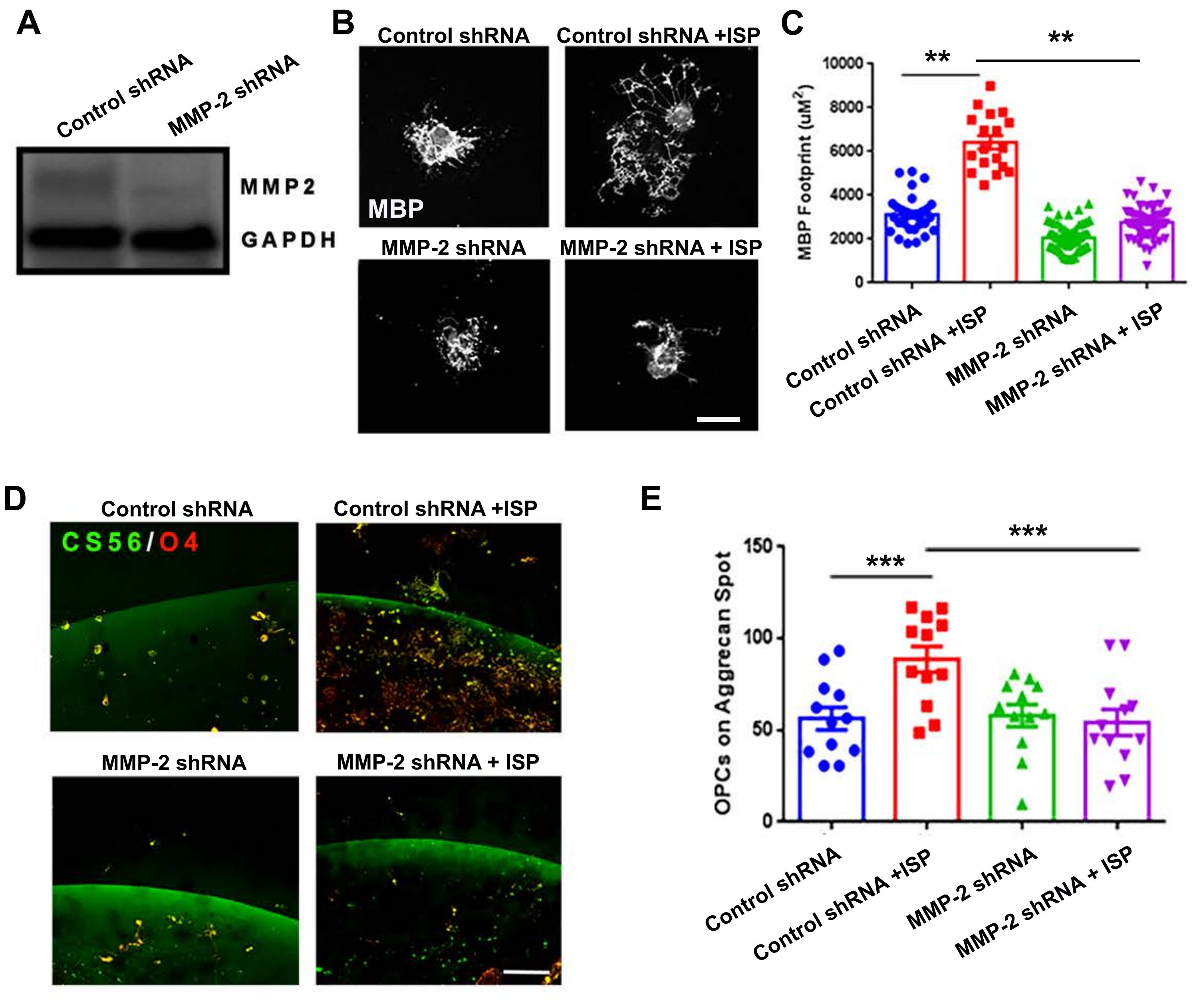

Supplementary Figure 10: shRNA knock down of MMP-2 decreases OPC maturation and migration on CSPGs to limit remyelination in cerebellar slices.

A. OPC cultures infected with control lentiviral scrambled shRNA or lentiviral particles expressing shRNA construct targeting MMP-2 for 48 hours before western blotting to assess MMP-2 knock down compared to GAPDH. **B-C.** Scrambled or shRNA targeting MMP-2 lentiviral-infected OPCs cultured on laminin and low concentration of aggrecan (1ug/mL) were immunostained with MBP following vehicle or ISP (2.5uM) treatment for 48 hours. Scale bar=100 µm.MBP area was quantified (One-Way ANOVA, Tukey's posthoc test, P=0.013, F(3, 158)=6.677, N=100 cells from 2 repeats). **D-E.** OPCs (O4, red) were infected with lentiviral constructs 48 hours before plating onto spot assays to assess migration across aggrecan (green) with or without ISP (2.5uM). Scale bar=100 µm. Number of OPCs crossing the aggrecan spot were counted (One-Way ANOVA, Tukey's posthoc test, P=0.0015, F(3, 44)=6.056. N=40 spots from 2 repeats). The data are presented as mean±s.e.m. **P<0.01, *** p < 0.001.

Supplementary Figure 11

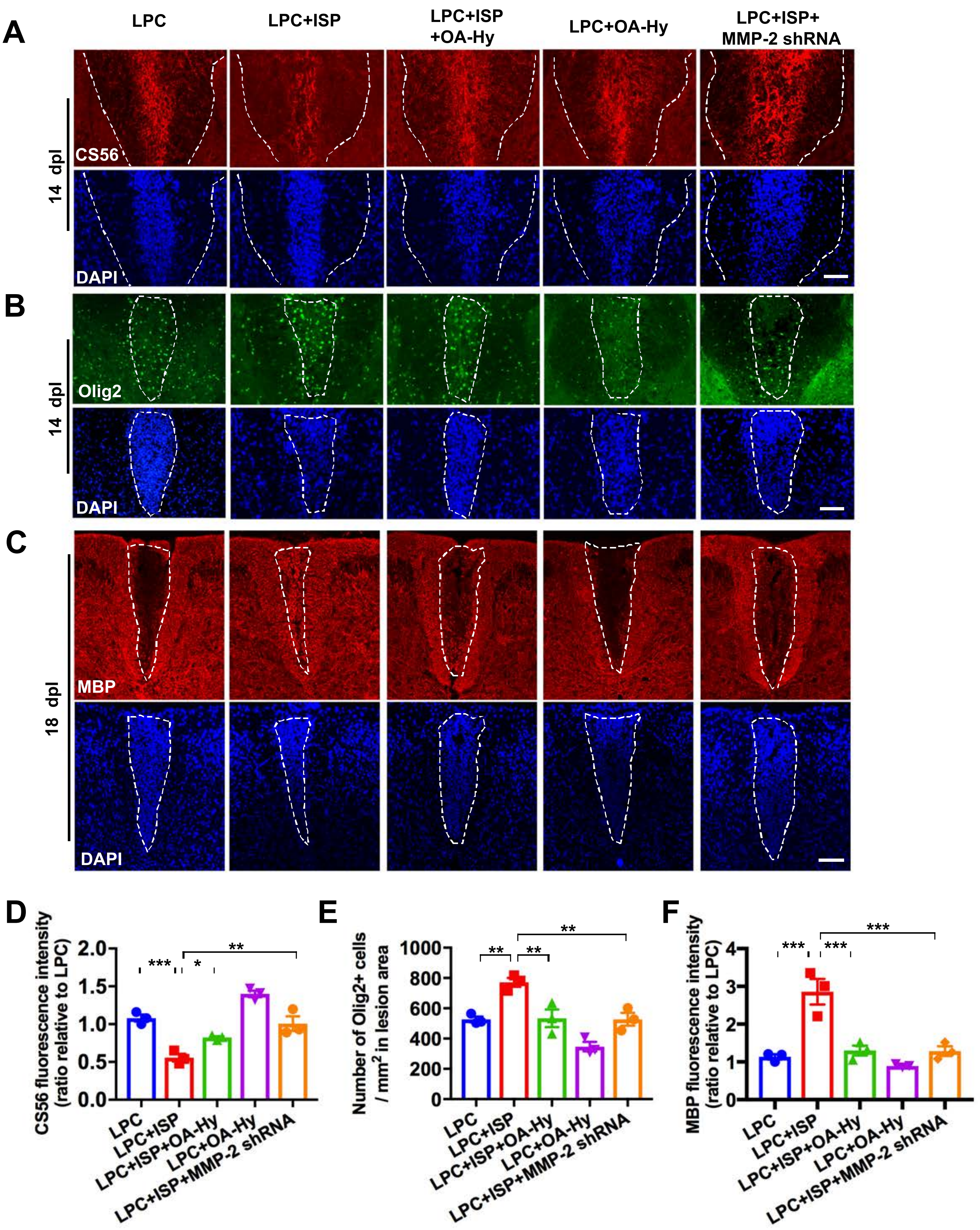

Supplementary Figure 11: MMP-2 mediates ISP-induced remyelination in LPC-demyelinated mouse model.

A. Representative images from immunohistochemistry of CS56 and DAPI from the spinal cords of vehicle, ISP, MMP-2 inhibitor (OA-Hy) and MMP-2 shRNA-treated mice show MMP-2-mediated CS56 degradation at 14 days post-LPC injection. Dashed lines demarcate dorsal white matter of spinal cord. Scare bar= 100 µm. **B.** Representative images from immunohistochemistry of Olig2 and DAPI from the spinal cords of vehicle, ISP, MMP-2 inhibitor (OA-Hy) and MMP-2 shRNA-treated mice show MMP-2-mediated OPCs migration at 14 days post-LPC injection. Dashed lines demarcate lesion areas. Scare bar= 100 µm. **C.** Representative images from immunohistochemistry of MBP and DAPI from the spinal cords of vehicle, ISP, MMP-2 inhibitor (OA-Hy) MMP-2 shRNA-treated mice show MMP-2-mediated myelin protein expression at 18 days post-LPC injection. Scare bar= 100 µm. **D.** Relative quantification of immunofluorescence intensity of CS56 in the spinal cord of vehicle, ISP, MMP-2 inhibitor (OA-Hy) and MMP-2 shRNA-treated mice at 14dpl (n=3 mice/group, ANOVA $F(4,10)=29.96$, Tukey's multiple comparison test, $P_{\text{LPC versus LPC+ISP}} = 0.0005$, $P_{\text{LPC+ISP versus LPC+ISP+OA-Hy}} = 0.0491$, $P_{\text{LPC +ISP versus LPC+ISP+MMP-2 shRNA}} = 0.0017$). **E.** Quantification of the number of Olig2^{+} cells in the spinal cord of vehicle, ISP, MMP-2 inhibitor (OA-Hy) and MMP-2 shRNA-treated mice at 14dpl (n=3 mice/group, ANOVA $F(4,10)=15.31$, Tukey's multiple comparison test, $P_{\text{LPC versus LPC+ISP}} = 0.008$, $P_{\text{LPC+ISP versus LPC+ISP+OA-Hy}} = 0.0097$, $P_{\text{LPC +ISP versus LPC+ISP+MMP-2 shRNA}} = 0.0082$). **F.** Relative quantification of immunofluorescence intensity of MBP in the dorsal spinal cord of vehicle, ISP, MMP-2 inhibitor (OA-Hy) or MMP-2 shRNA-treated mice at 18dpl (n=3 mice/group, ANOVA $F(4,10)=19.79$, Tukey's multiple comparison test, $P_{\text{LPC versus LPC+ISP}} = 0.0003$, $P_{\text{LPC+ISP versus LPC+ISP+OA-Hy}} = 0.0007$, $P_{\text{LPC +ISP versus LPC+ISP+MMP-2 shRNA}} = 0.0006$). The data are presented as mean±s.e.m. $^{*}P<0.05$, $^{**}P<0.01$, $^{***}p < 0.001$.

Supplementary Figure 12

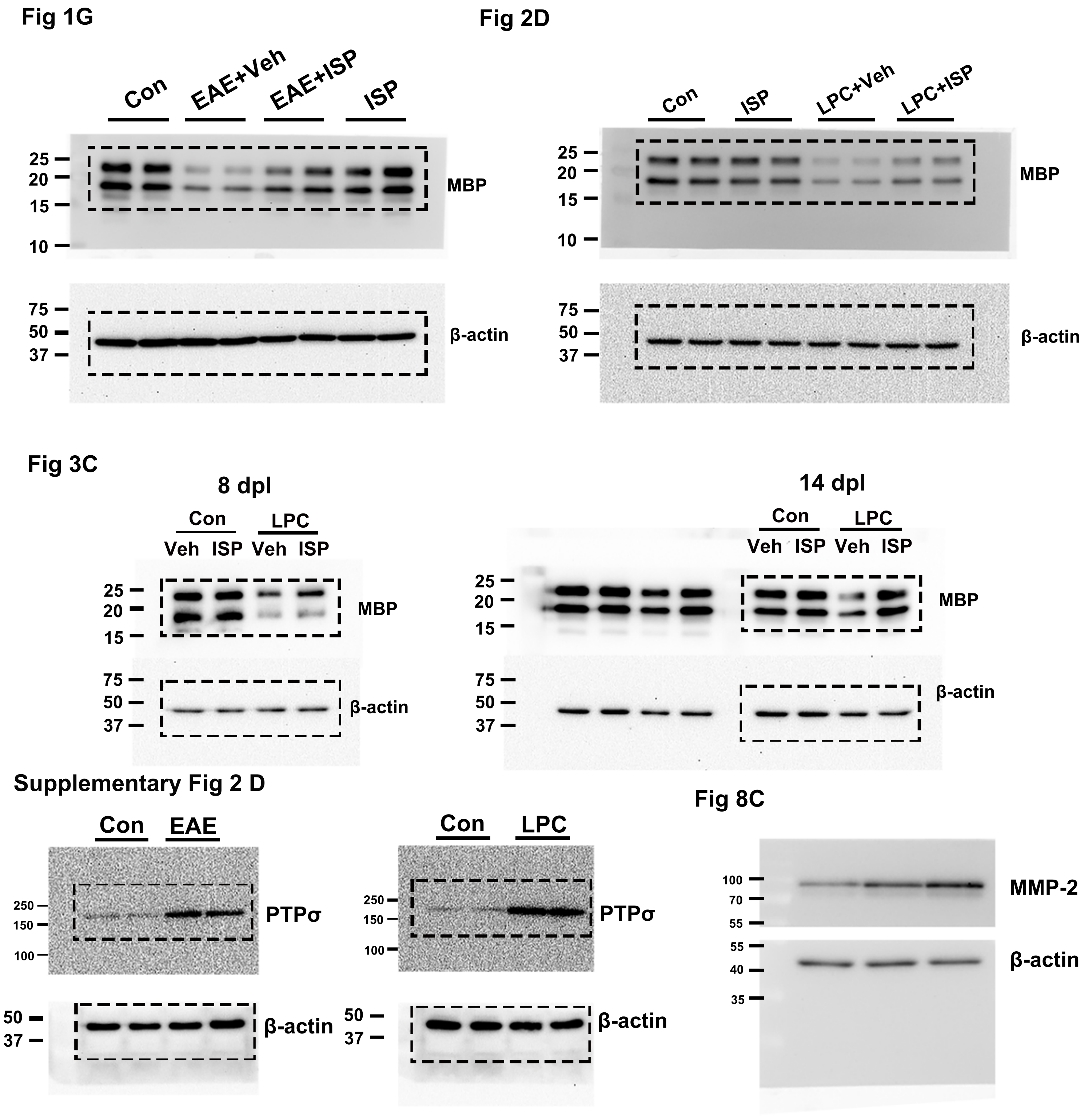

Supplementary Figure 12. Full-length images of blots in Figures 1, 2, 3 and supplementary Figures 2, 8.

Supplementary Figure 13

Fig 5C

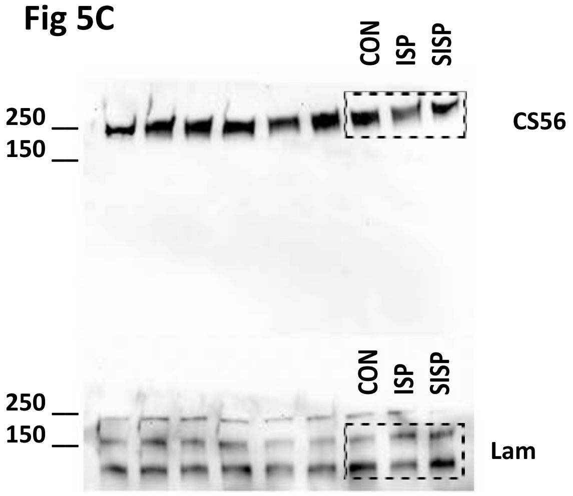

Fig 6C

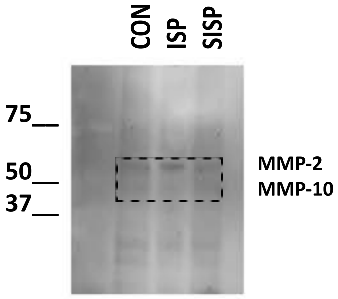

Fig 6I

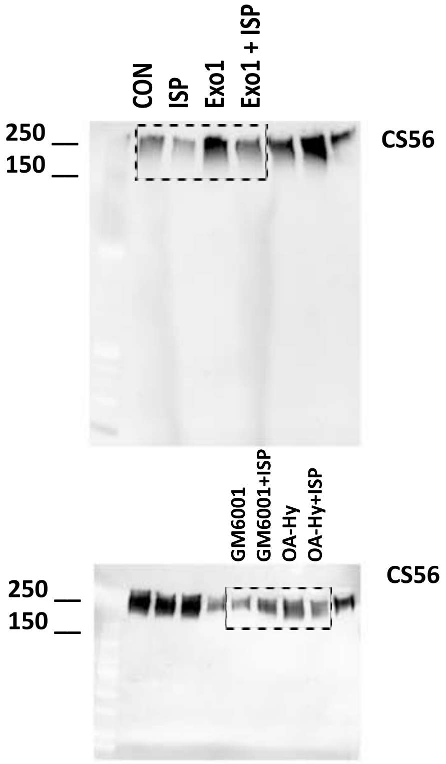

Fig 6F

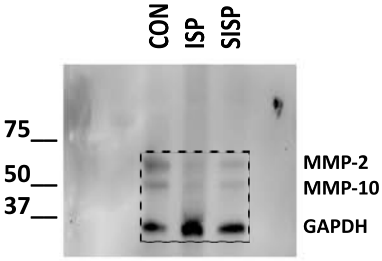

Supplementary Fig 7G

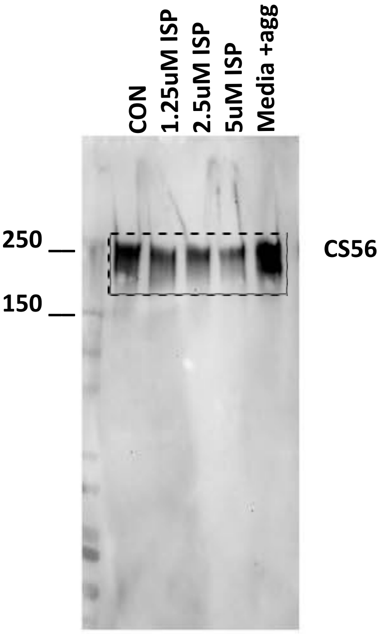

Supplementary Fig 10A

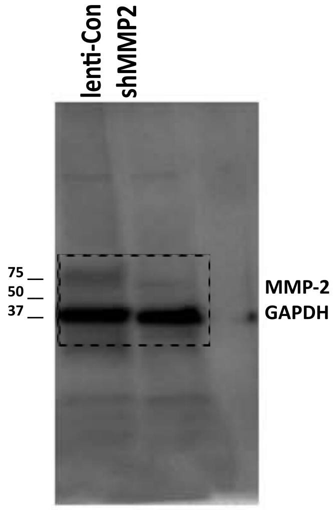

Supplementary Figure 13. Full-length images of blots in Figures 5, 6 and supplementary Figure 7, 10.
